# Supplementary material for: Transcriptome Analysis Reveals Vimentin-Induced Disruption of Cell–Cell Associations Augments Breast Cancer Cell Migration
Source: Cells. 2022 Dec 13;11(24):4035. doi: 10.3390/cells11244035 (PMC9776984; doi:10.3390/cells11244035)
Supplement: Supplementary file 1 [file cells-11-04035-s001.zip › DEGs and enrichment analysis.zip!/DEGs and enrichment analysis/WT_VIMvsVector GO enrichment/UP/WT_VIMvsVector_up.GObp_DAG.pdf]

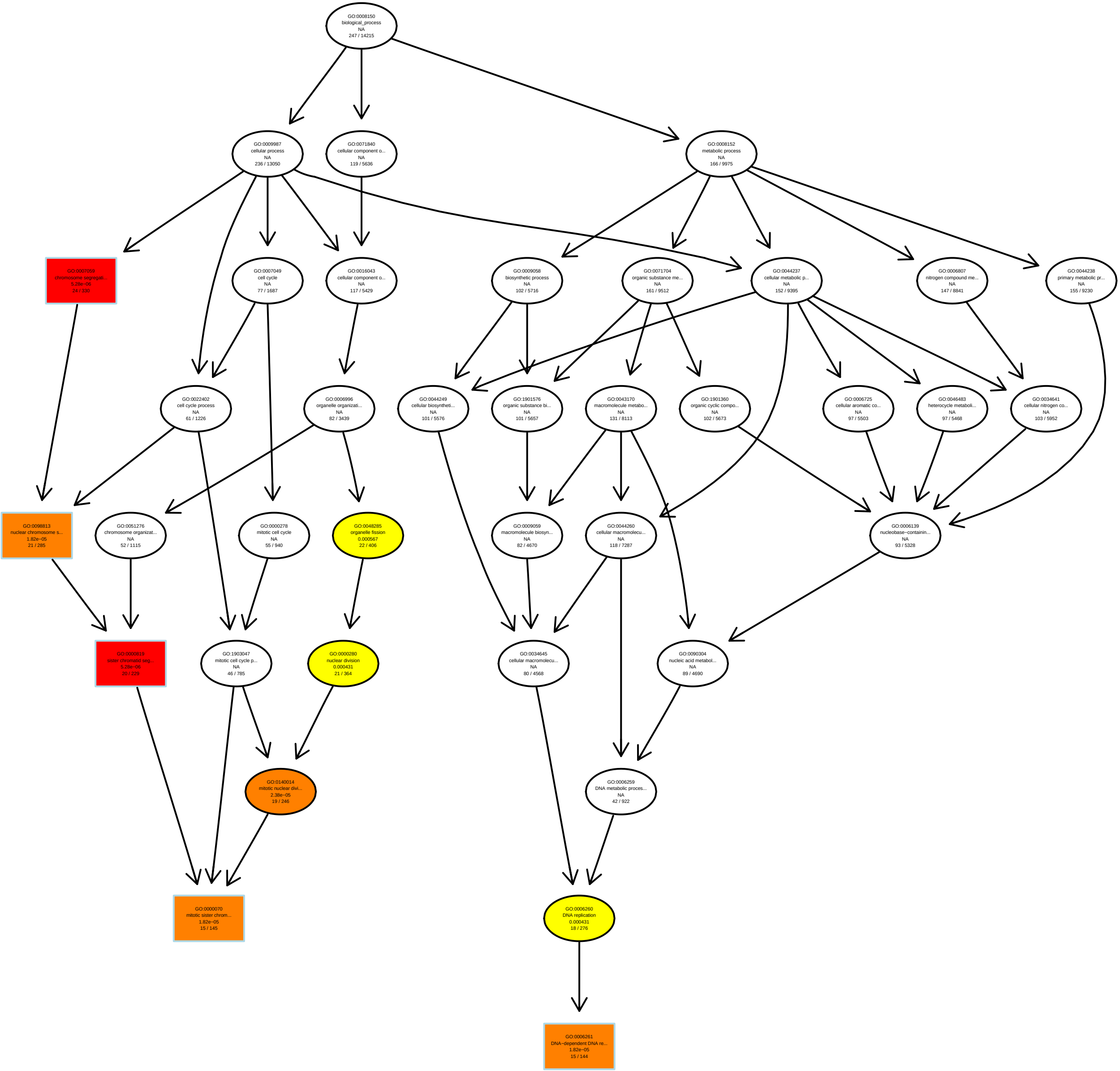
GO:0008150
biological_process
NA
247 / 14215
GO:0009987
cellular process
NA
236 / 13050
GO:0071840
cellular component o...
NA
119 / 5636
GO:0008152
metabolic process
NA
166 / 9975
GO:0007059
chromosome segregati...
5.28e−06
24 / 330
GO:0007049
cell cycle
NA
77 / 1687
GO:0016043
cellular component o...
NA
117 / 5429
GO:0009058
biosynthetic process
NA
102 / 5716
GO:0071704
organic substance me...
NA
161 / 9512
GO:0044237
cellular metabolic p...
NA
152 / 9395
GO:0006807
nitrogen compound me...
NA
147 / 8841
GO:0044238
primary metabolic pr...
NA
155 / 9230
GO:0022402
cell cycle process
NA
61 / 1226
GO:0006996
organelle organizati...
NA
82 / 3439
GO:0044249
cellular biosyntheti...
NA
101 / 5576
GO:1901576
organic substance bi...
NA
101 / 5657
GO:0043170
macromolecule metabo...
NA
131 / 8113
GO:1901360
organic cyclic compo...
NA
102 / 5673
GO:0006725
cellular aromatic co...
NA
97 / 5503
GO:0046483
heterocycle metaboli...
NA
97 / 5468
GO:0034641
cellular nitrogen co...
NA
103 / 5952
GO:0098813
nuclear chromosome s...
1.82e−05
21 / 285
GO:0051276
chromosome organizat...
NA
52 / 1115
GO:0000278
mitotic cell cycle
NA
55 / 940
GO:0048285
organelle fission
0.000567
22 / 406
GO:0009059
macromolecule biosyn...
NA
82 / 4670
GO:0044260
cellular macromolecu...
NA
118 / 7287
GO:0006139
nucleobase−containin...
NA
93 / 5328
GO:0000819
sister chromatid seg...
5.28e−06
20 / 229
GO:1903047
mitotic cell cycle p...
NA
46 / 785
GO:0000280
nuclear division
0.000431
21 / 364
GO:0034645
cellular macromolecu...
NA
80 / 4568
GO:0090304
nucleic acid metabol...
NA
89 / 4690
GO:0140014
mitotic nuclear divi...
2.38e−05
19 / 246
GO:0006259
DNA metabolic proces...
NA
42 / 922
GO:0000070
mitotic sister chrom...
1.82e−05
15 / 145
GO:0006260
DNA replication
0.000431
18 / 276
GO:0006261
DNA−dependent DNA re...
1.82e−05
15 / 144
